# Positive association between Cheyne-Stokes respiration events and diastolic dysfunction in pre-heart failure: a cross-sectional study with longitudinal implications

**DOI:** 10.3389/fcvm.2025.1607079

**Published:** 2025-09-25

**Authors:** Cai Qinghao, Yang Yifan, Ouyang Lijun, Chen Yahui, Yang Zhimin, Xu Biyun

**Affiliations:** ^1^ State Key Laboratory of Traditional Chinese Medicine Syndrome, Department of Sleep Medicine, Guangzhou, Guangdong, China; ^2^ The Second Affiliated Hospital of Guangzhou University of Chinese Medicine (Guangdong Provincial Hospital of Chinese Medicine), Guangzhou, Guangdong, China; ^3^ Chinese Medicine Guangdong Laboratory (Hengqin Laboratory), Zhuhai, China

**Keywords:** sleep-related breathing disorders, Cheyne-Stokes respiration, diastolic dysfunction, pre-heart failure, HFPEF

## Abstract

**Background:**

Cheyne-Stokes respiration (CSR), a distinct type of sleep-related breathing disorder, is closely associated with heart failure (HF). In clinical practice, it has been observed that some patients with CSR do not present with HF or related symptoms. However, limited studies have investigated this phenomenon. This study aimed to explore whether CSR events may indicate specific cardiac structural/functional alterations or serve as an early warning sign for HF progression.

**Materials and methods:**

We enrolled middle-aged and elderly patients (≥45 years) hospitalized at Guangdong Provincial Hospital of Traditional Chinese Medicine without a diagnosis or symptoms of HF. Data on medical history, echocardiography, BNP/NT-proBNP levels, and polysomnography were collected. Participants were categorized into three groups based on PSG results: (1) Sleep-Related breathing disorders with CSR, (2) Sleep-Related breathing disorders without CSR, and (3) no Sleep-Related breathing disorders. Comparative analyses of clinical parameters were performed across groups.

**Results:**

A total of 171 patients were included. Patients with CSR events exhibited significantly higher BNP/Nt-proBNP levels and more pronounced cardiac structural remodeling, including left atrial enlargement and elevated E/e' ratios. Further analysis identified CSR events as independent risk factors for elevated BNP/Nt-proBNP levels [OR = 2.02, 95%CI (1.02–3.98), *p* = 0.044], left atrial diameter index [OR = 3.15, 95%CI (1.50–6.64), *p* = 0.002], and E/e’ ratio [OR = 15.32, 95%CI (6.48–36.22), *p* < 0.001].

**Conclusion:**

In patients without overt HF, the presence of CSR events is positively correlated with left ventricular diastolic dysfunction. CSR events may serve as a biomarker for persistent cardiac diastolic impairment or an early indicator of pathological progression toward heart failure with preserved ejection fraction (HFpEF). These findings warrant further longitudinal investigations to validate its predictive value in clinical settings.

## Introduction

In sleep-related breathing disorders (SBD), Cheyne-Stokes respiration (CSR) represents a distinct form of central sleep apnea event. Current research has demonstrated its association with heart failure (HF), atrial fibrillation, renal diseases, and neurological disorders, with particularly extensive studies focusing on its relationship with congestive heart failure (1). However, current understanding of central sleep apnea (CSA) and CSR remains limited, particularly regarding their underlying mechanisms and clinical implications.

Most studies suggest that the combination of CSA with CSR plays an important role in the progression of heart function deterioration and is a strong independent indicator of the mortality rate of patients with atrial fibrillation (AF) and heart failure (2–8). May (6) demonstrated that CSR serves as an independent predictor of AF onset, particularly among older participants. Calvin (3) found that Heart failure patients with CSA and CSR exhibited left atrial enlargement and were older compared to those without CSA. Lo (4) found that CSR were associated with lower ejection fraction and larger left atrial size. Bitter (5) found that in patients with heart failure and preserved ejection fraction (HFpEF), cases of CSA, as a proportion of SBD, increased alongside increases in the degree of impairment of left ventricular diastolic function. Saito (7, 8) further validated the correlation between CSR and both AF and HF during long-term continuous positive airway pressure (CPAP) monitoring, and proposed that tracking CSR changes may facilitate early-stage prediction of HF onset and deterioration. Alternative perspectives propose that CSA with CSR may be a compensatory response to impending heart failure, and CSR could be a protection mechanism in HF (9, 10).

Besides, Most of the aforementioned studies were conducted using an unattended ambulatory system rather than standard polysomnography (PSG) to screen for Obstructive Sleep Apnea (OSA) or CSA and did not further identify or discuss the impact of respiratory events. For example, although mixed apnea events are classified under the obstructive sleep apnea index (OAHI), their potential inclusion of CSR (11, 12) has rarely been discussed in clinical contexts.

While existing studies on SBD with CSR have predominantly targeted advanced heart failure populations, research in patients without a confirmed diagnosis of heart failure remains limited. Notably, CSR events have been clinically documented in patients with neither a confirmed diagnosis of heart failure nor related symptoms.

Therefore, we propose to analyze echocardiography, BNP/NT-proBNP level, PSG and medical history in patients without a confirmed diagnosis of heart failure and presenting no related symptoms, with the aim of investigating whether CSR events correlate with specific cardiac structural/functional alterations and potentially indicate occult heart failure states.

## Materials and methods

This was a cross-sectional study that collected data including medical history, PSG and echocardiography from hospitalized patients from Jan. 2018 to Jan. 2025 at Guangdong Provincial Hospital of Traditional Chinese Medicine. This retrospective analysis was granted exemption by the Guangdong Provincial Hospital of Chinese Medicine Ethics Committee, as it utilized de-identified data from a parent study with existing informed consent.

Inclusion criteria:

1. Patients aged ≥45 years.
2. No documented history of acute or chronic heart failure, and absence of current clinical manifestations associated with heart failure, including: Dyspnea at rest or exertional breathlessness; Unexplained fatigue during daily activities; Lower limb edema not attributable to venous insufficiency or renal dysfunction.
3. BNP levels <500 ng/L or NT-proBNP levels below age-adjusted thresholds: <450 ng/L for individuals aged <50 years, <900 ng/L for those aged 50–75 years, and <1,800 ng/L for those aged >75 years.
4. PSG results confirming SBD based on the International Classification of Sleep Disorders, 3rd Edition (ICSD-3) criteria.

Exclusion criteria:

1. Patients diagnosed with acute cerebrocardiovascular diseases or metabolic disorders during hospitalization, or those administered morphine-related medications that may induce short-term CSR.
2. Undefined SBD according to ICSD-3 diagnostic criteria, including failure to meet quantitative thresholds (e.g., 5 ≤ AHI < 15 and absence of clinical symptoms).
3. Current use of continuous positive airway pressure (CPAP) therapy for sleep apnea management.

Groups:

1. Group with SBD and CSR Events (SBD + CSR): Patients meeting diagnostic criteria for SBD (AHI ≥ 15 or AHI ≥ 5 with clinical symptoms), and exhibiting CSR events confirmed by PSG.
2. Group with SBD without CSB Event (SBD-CSR): Patients meeting diagnostic criteria for SBD, absence of CSR event and no consecutive mixed apnea episodes (combined obstructive and central apneas ≥10 s, ≥3 events with a cycle length of ≥40 s) confirmed by PSG.
3. Group without SBD (NonSBD): Patients not meeting diagnostic criteria for SBD (AHI < 5), absence of CSR event and no consecutive mixed apnea episodes confirmed by PSG.

### Polysomnography

The PSG test used Type 2 sleep testing devices including an electroencephalogram, electrooculography and mandibular electromyography. Sleep stages and respiratory events were scored according to the AASM 2.3 recommended rules (13). Mixed Apnea was defined as an apnea with absent inspiratory effort in the initial part of the event, followed by a resumption of inspiratory effort in the second part of the event. Hypopnoea was defined as a 30% decrease in pressure airflow from baseline, accompanied by arousal or oxygen desaturation ≥3%. A CSR events was defined as episodes of ≥3 consecutive central apneas and/or central hypopneas, separated by a crescendo and decrescendo change in breathing amplitude such as CSR with a cycle length of ≥40 s. CSR duration index (CSR/TST%) was defined as all CSR events duration divided by TST.

### Echocardiography

Diameters of the left atrium and left ventricle were measured by M-Mode echocardiography and corrected by 2D echocardiography following the guidelines set by the American Society of Echocardiography (ASE) (14). All abnormalities of echocardiography parameters are based on the recommended by ASE guidelines. Cardiac structure was evaluated through the left atrial antero-posterior diameter (LAd), right ventricular internal dimension (RVd), left ventricular end-systolic dimension (LVIDs), left ventricular end-diastolic dimension (LVIDd), left ventricular posterior wall thickness (LVPW), interventricular septal thickness at diastole (IVSd) and pulmonary artery diameter (PA); left atrial diameter index (LADi) was calculated through dividing LAd by body surface area (BSA); Left ventricular ejection fraction (LVEF%) evaluated left ventricular systolic function; Average E/e' ratios were collected to evaluate left ventricular diastolic function.

### Definition of BNP/NT-proBNP levels

BNP and NT-proBNP levels were categorized into three clinical tiers based on established heart failure diagnostic thresholds (15):

1. Normal range (BNP < 35 pg/ml, NT-proBNP < 125 pg/ml), applicable to individuals without evidence of acute or chronic heart failure.
2. Mildly elevated (35 pg/ml ≤ BNP < 100 pg/ml, 125 pg/ml ≤ NT-proBNP < 300 pg/ml), which may require serial monitoring but does not confirm heart failure diagnosis.
3. Significantly elevated (100 pg/ml ≤ BNP < 500 pg/ml, 300 pg/ml ≤ NT-proBNP < 450–1,800 pg/ml with age-stratified thresholds), indicating increased risk of heart failure.

To account for the correlation between biomarkers, a logarithmic transformation model was applied according to Hamatani, et al. (16), defined as: log (NT-proBNP) = 1.1 × log(BNP) + 0.570. This standardization enabled parametric linear regression analysis of relationships.

### Statistics

Counting data was expressed by rate (%), for normally distributed measurement data, the mean ± standard deviation was used, and for non-normally distributed data, the median (P25, P75) was used. The baseline demographic characteristics and echocardiography characteristics of the four groups were compared by using Pearson’s chi-squared test for categorical variables and the Kruskal–Wallis test for continuous variables. Significant variables identified in univariate screening were further analyzed using logistic regression and linear regression, with adjustments for covariates. Statistical analyses were performed by using SPSS ver. 26.0. For all analyses, *p* < 0.05 was regarded as significant.

## Result

This study enrolled a total of 171 patients. Group SBD + CSR demonstrated significantly higher mean age (*χ*^2^ = 24.687, *p* < 0.001) and prevalence of Af (*χ*^2^ = 11.078, *p* = 0.004) compared to the other groups. In contrast, Group nonSBD exhibited a markedly lower mean BMI (*χ*^2^ = 12.839, *P* = 0.002) and prevalence of HBP (*χ*^2^ = 20.182, *p* < 0.001) relative to the remaining cohorts. Of the 78 patients who completed the 2-year follow-up, 20% (4/20) in Group SBD + CSR were clinically diagnosed with heart failure during the follow-up period (Table 1).

**Table 1:** Patient information.

Characteristic	SBD + CSR (*n* = 45)	SBD-CSR (*n* = 85)	NonSBD (*n* = 41)	Statistical analysis
Gen(M:F)	27:18	58:27	23:18	*χ*^2^ = 2.013, *p* = 0.366
Age	69.0 ± 10.2	60.5 ± 9.7	58.8 ± 8.9	*χ*^2^ = 24.687, *P* < 0.001 SBD + CSR > (SBD-CSR, NoSBD)
BMI	27.0 ± 3.8	27.0 ± 3.5	24.7 ± 3.3	*χ*^2^ = 12.839, *P* = 0.002 SBD > NoSBD
AHI	36.5 (22.8, 50.6)	33.9 (21.7, 49.8)	2.7 (1.3, 3.4)	
OAHI	24.6 (13.2, 40.3)	30.8 (20.9, 48.1)	2.0 (1.0, 3.0)	
CAHI	8.5 (6.1, 15.4)	0.9 (0.1, 2.9)	0.1 (0, 0.3)	
MAI	1.5 (0.1, 3.9)	0.5 (0, 2.8)	0 (0, 0)	
CSR Event duration (min)	24.2 (11.8, 38.3)	–	–	
Mean SpO2	94.0 (92.0, 95.0)	94.0 (93.0, 95.0)	95.0 (94.0, 96.0)	
Lowest SpO2	81.0 (75.0, 85.0)	81.0 (74.0, 84.0)	89.0 (87.0, 91.0)	
SpO2 < 90% duration (min)	18.4 (6.0, 66.7)	22.2 (8.3, 5 6.4)	0.2 (0, 1.2)	
Total sleep time	382.0 ± 79.0	391.0 ± 81.0	391.9 ± 78.4	
Medical history				
HBP	40 (88.9%)	64 (75.3%)	19 (46.3%)	*χ*^2^ = 20.182, *p* < 0.001 SBD > NoSBD
Af	7 (15.6%)	3 (3.5%)	0	*χ*^2^ = 11.078, *p* = 0.004 SBD + CSR > (SBD-CSR, NoSBD)
CHD	19 (42.2%)	27 (31.8%)	9 (22.0%)	*χ*^2^ = 4.053, *p* = 0.132
PD	5 (11.1%)	2 (2.4%)	3 (7.3%)	*χ*^2^ = 4.310, *p* = 0.116
CVD	10 (22.2%)	9 (10.6%)	5 (12.2%)	*χ*^2^ = 3.452, *p* = 0.178
CKD	4 (8.9%)	3 (3.5%)	2 (4.9%)	*χ*^2^ = 1.711, *P* = 0.452
Clinical HF was confirmed over a two-year follow-up	4/20 (20%)	0/41 (0%)	0/17 (0%)	*χ*^2^ = 12.227, *p* = 0.002 SBD + CSR > SBD-CSR

SBD + CSR, sleep breathing disorder with CSR Events; SBD-CSR, sleep breathing disorder without CSR Events; NonSBD, patients without Sleep breathing disorder.

BMI, mass body mass index; AHI, apnea hypopnea index; HBP, high blood pressure; Af, atrial fibrillation; CHD, coronary heart disease; PD, Parkinson's disease; CVD, cerebrovascular diseases; CKD, chronic kidney disease; HF, heart failure.

**Figure 1:**
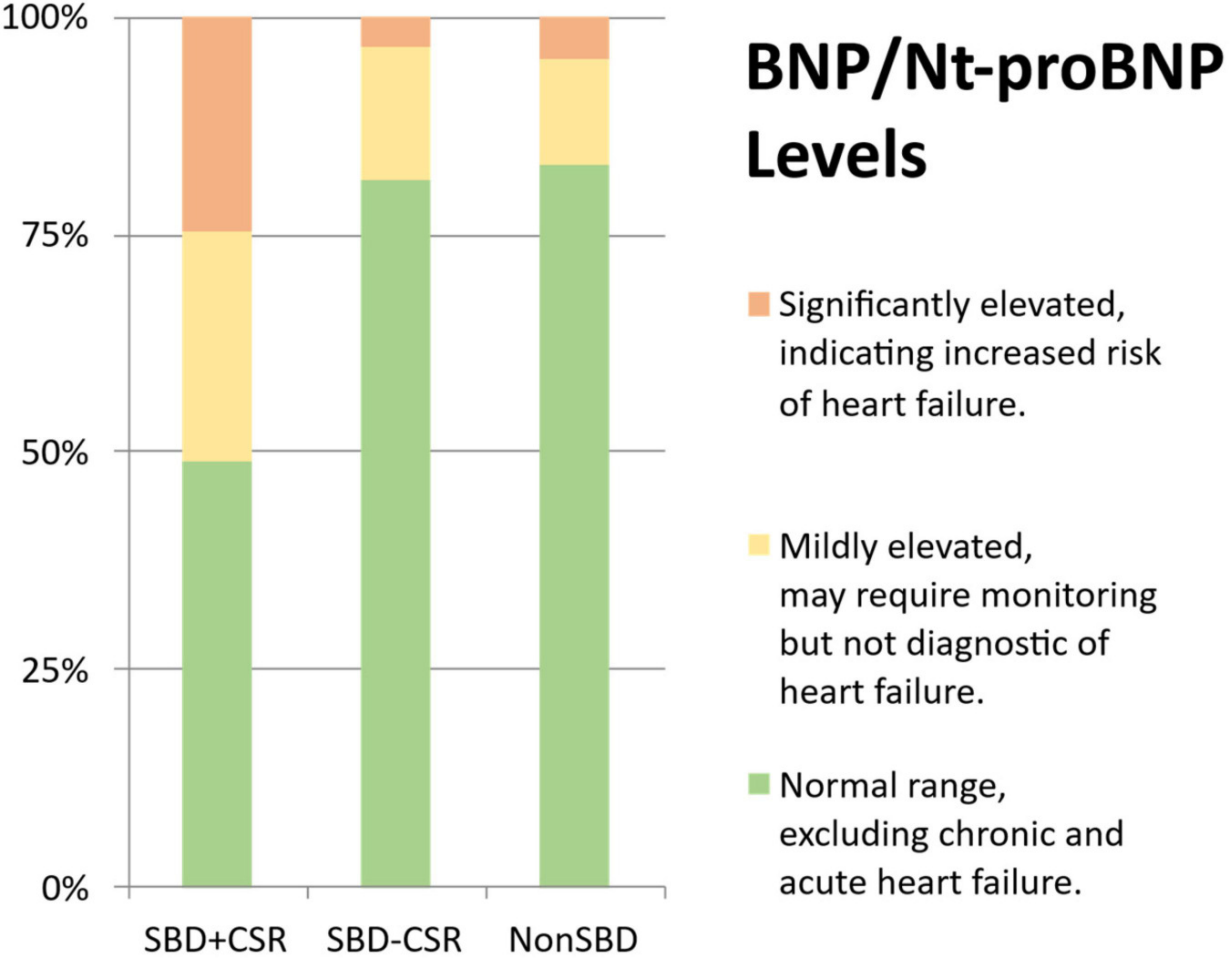
BNP/NT-proBNP level. **(a)** Normal range (BNP < 35 pg/ml, NT-proBNP < 125 pg/ml); **(b)** mildly elevated (35 pg/ml ≤ BNP < 100 pg/ml, 125 pg/ml ≤ NT-proBNP < 300 pg/ml); **(c)** significantly elevated (100 pg/ml ≤ BNP < 500 pg/ml, 300p g/ml ≤ NT-proBNP < 450–1,800 pg/ml with age-stratified thresholds).

**Figure 2:**
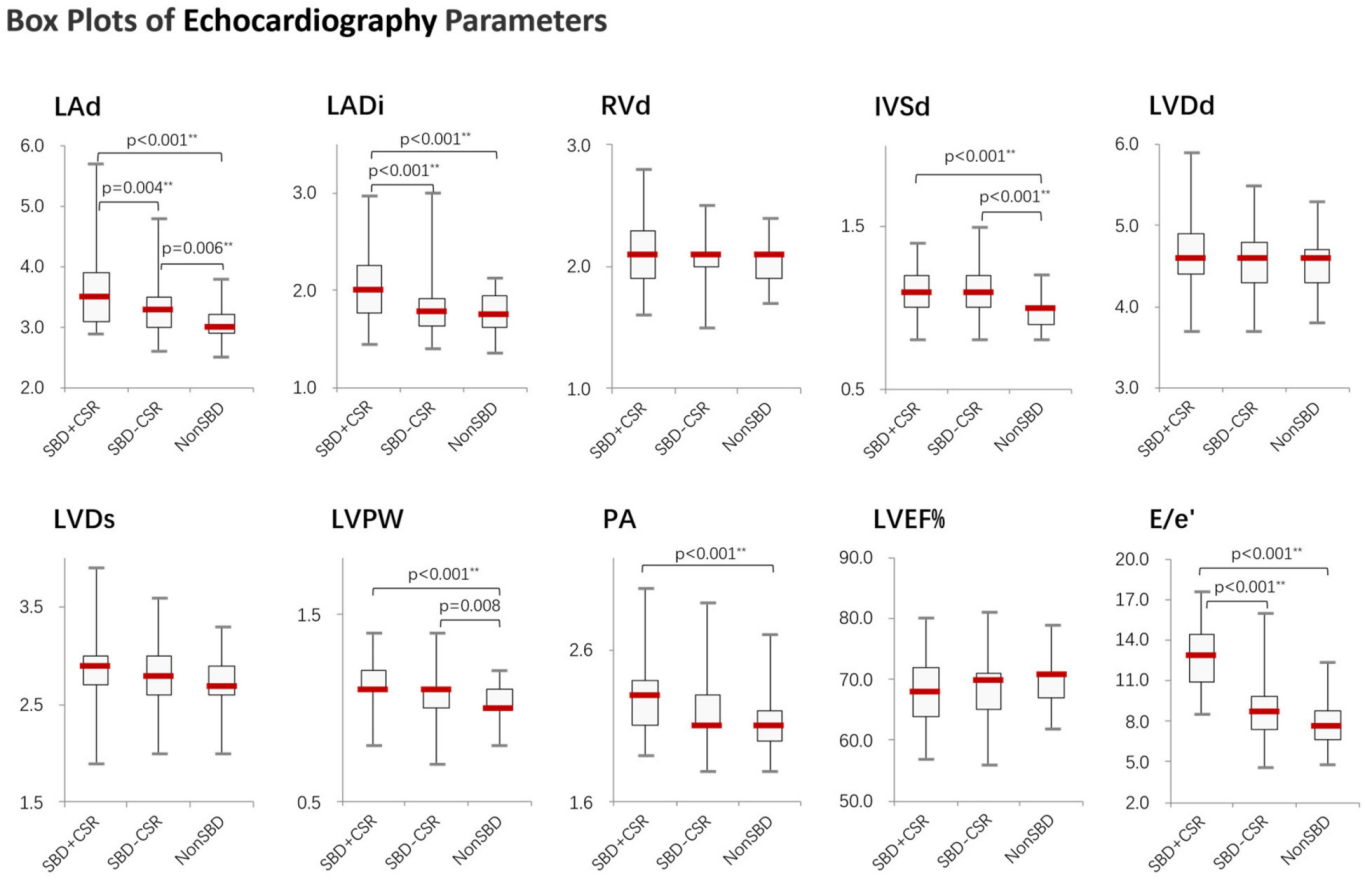
Boxplots showing the distribution of echocardiography parameters across study groups. Significant differences between groups were determined by the Kruskal–Wallis test followed by Mann–Whitney *U* test, applying a Bonferroni correction to adjust the alpha level for multiple comparisons (*p < 0.05, **p < 0.01). SBD + CSR = Sleep breathing disorder with CSR events; SBD-CSR, sleep breathing disorder without CSR events; NonSBD, patients without sleep breathing disorder.

Significant differences were observed across groups in both BNP/Nt-proBNP levels (Figure 1 and Supplementary Table 1) and structural cardiac parameters (Table 2). Group SBD + CSR exhibited markedly higher BNP/Nt-proBNP levels compared to Groups SBD-CSR and nonSBD (*χ*^2^ = 22.869, *p* < 0.001, Figure 1). Echocardiographic analysis further revealed that Group SBD + CSR had larger left atrial dimensions (LAd: 3.5[3.1, 3.9] cm vs. 3.3[2.9, 3.5]/3.0[2.9, 3.2] cm in Group SBD-CSR/nonSBD, *p* = 0.004/*p* < 0.001; LADi: 2.0[1.8, 2.3] cm/m² vs. 1.8[1.6, 1.9]/1.8[1.6, 2.0] cm/m^2^ in Group SBD-CSR/nonSBD, *p* < 0.001/*p* < 0.001) and higher average E/e' ratios [12.9[10.7, 14.5] vs. 8.7[7.4, 9.8]/7.6[6.7, 8.8] in Group SBD-CSR/nonSBD, *p* < 0.001/*p* < 0.001]. Conversely, Group nonSBD demonstrated reduced IVSd [1.0[0.9, 1.1] vs. 1.1[1.0, 1.2]/1.1[1.0, 1.2] in Group SBD + CSR/SBD-CSR, *p* < 0.001/*p* < 0.001] and LVPW [1.0[1.0, 1.1] vs. 1.1[1.1, 1.2]/1.1[1.0, 1.1] in Group SBD + CSR/SBD-CSR, *p* < 0.001/*p* = 0.008] relative to other groups (Figure 2).

**Table 2:** Echocardiography parameters (length: cm) by Kruskal–Wallis test.

Param.	SBD + CSR (*n* = 45)	SBD-CSR (*n* = 85)	NonSBD (*n* = 41)	*χ* ^2^	*p*
LAd	3.5 (3.1, 3.9)	3.3 (2.9, 3.5)	3.0 (2.9, 3.2)	26.394	**<0**.**001**
LADi	2.0 (1.8, 2.3)	1.8 (1.6, 1.9)	1.8 (1.6, 2.0)	19.930	**<0**.**001**
RVd	2.1 (1.9, 2.3)	2.1 (2.0, 2.1)	2.1 (1.9, 2.2)	0.191	0.909
IVSd	1.1 (1.0, 1.2)	1.1 (1.0, 1.2)	1.0 (0.9, 1.1)	22.847	**<0**.**001**
LVIDd	4.6 (4.4, 4.9)	4.6 (4.3, 4.8)	4.6 (4.3, 4.8)	0.341	0.843
LVIDs	2.9 (2.7, 3.0)	2.8 (2.6, 3.0)	2.7 (2.6, 2.9)	3.661	0.160
LVPW	1.1 (1.1, 1.2)	1.1 (1.0, 1.1)	1.0 (1.0, 1.1)	17.618	**<0**.**001**
PA	2.3 (2.1, 2.4)	2.1 (2.1, 2.3)	2.1 (2.0, 2.2)	14.347	**0**.**001**
LVEF%	68.0 (63.5, 72.0)	70.0 (65.0, 71.5)	71.0 (66.5, 71.5)	2.156	0.340
E/e’	12.9 (10.7, 14.5)	8.7 (7.4, 9.8)	7.6(6.7, 8.8)	68.597	**<0**.**001**

SBD + CSR, sleep breathing disorder patients with CSR events; SBD-CSR, sleep breathing disorder patients without CSR Events; NonSBD, patients without sleep breathing disorder.

Bold values indicate statistical significance at *p* < 0.05.

Ordinal logistic regression analysis of variables associated with increased BNP/Nt-proBNP levels revealed that age [OR = 1.09 per year, 95%CI (1.05–1.13), *p* < 0.001], CSR Events [OR = 2.02, 95%CI (1.02–3.98), *p* = 0.044], lowest SpO2 [OR = 0.93, 95%CI (0.86–1.00), *p* = 0.044] and atrial fibrillation [OR = 5.11, 95%CI (1.96–13.32), *p* = 0.001] were independent predictors (Table 3). Analysis of echocardiography parameters, LAd revealed consistent associations for age [OR = 1.07 per year, 95%CI (1.04–1.10), *p* < 0.001], BMI [OR = 1.15, 95%CI (1.05–1.26), *p* = 0.003] and atrial fibrillation [OR = 6.18, 95%CI (1.11–34.44), *p* = 0.038] (Table 4). Extending this framework to LADi categories demonstrated overlapping risk profiles: age [OR = 1.08 per year, 95%CI (1.05–1.12)] and BMI [OR = 0.90(0.83–0.99)] maintained significance, with left atrial diameter index uniquely linked to CSR Events [OR = 3.15, 95%CI (1.50–6.64), *p* = 0.002] (Table 5). Similar patterns emerged in average E/e' analysis for age [OR = 1.05 per year, 95%CI (1.01–1.08), *p* = 0.006] and CSR Events [OR = 15.32, 95%CI (6.48–36.22), *p* < 0.001] (Table 6). Separate parallel lines tests for all model confirmed the proportional odds assumption, ensuring consistent coefficient interpretation across outcome categories.

**Table 3:** Ordinal logistic regression analysis results of BNP/Nt-proBNP level.

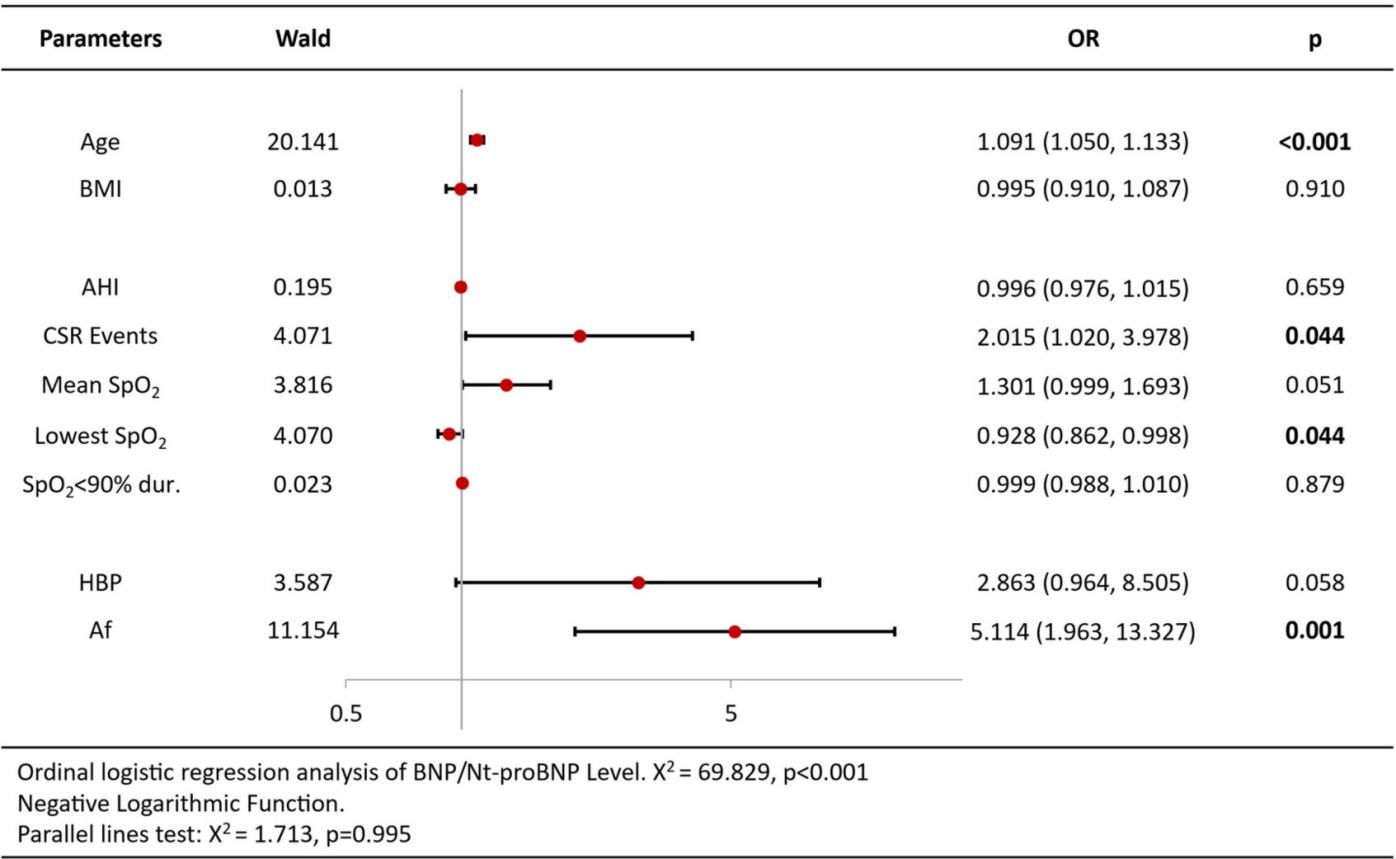			

**Table 4:** Ordinal logistic regression analysis results of left atrial antero-posterior diameter.

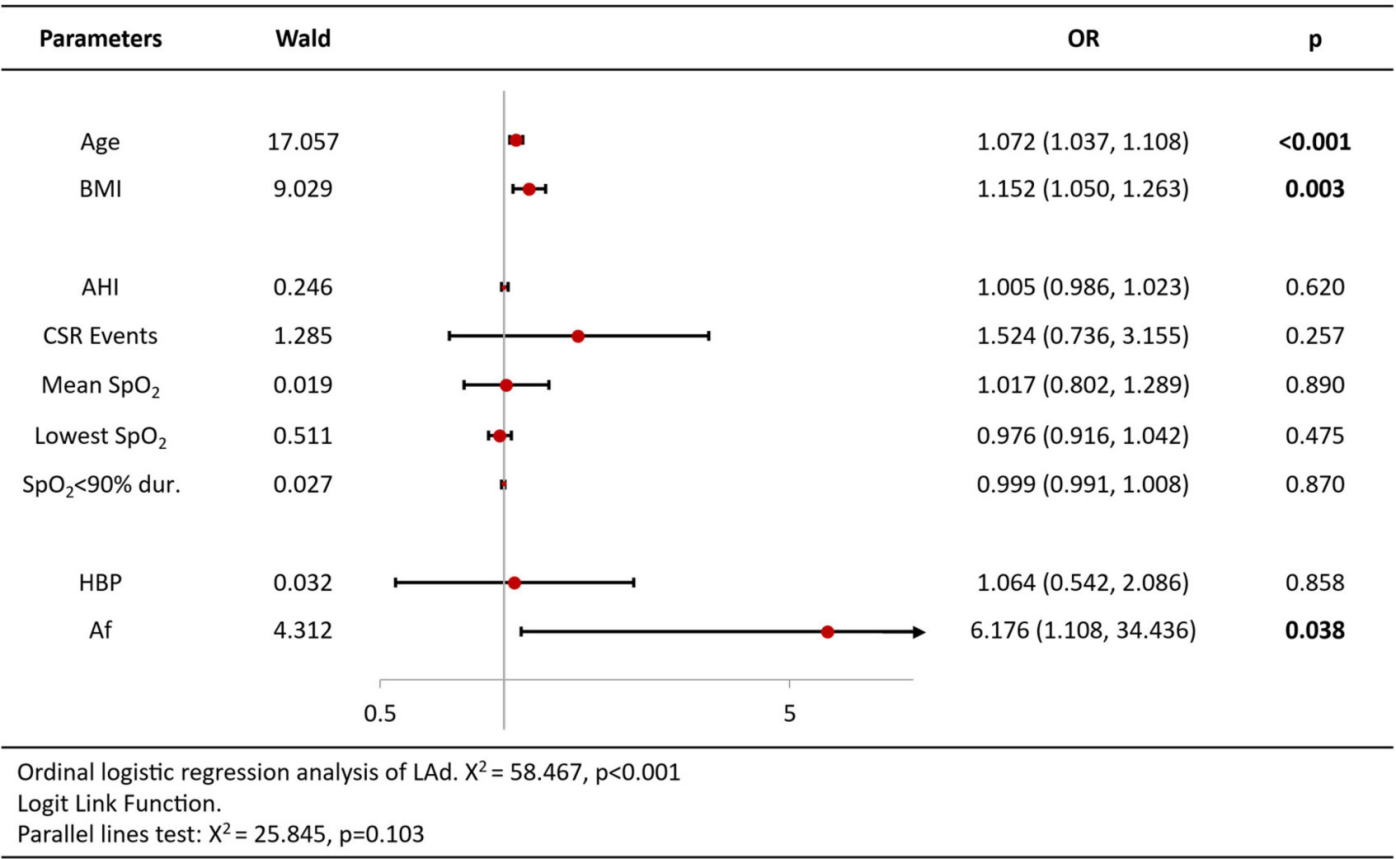			

**Table 5:** Ordinal logistic regression analysis results of LADi.

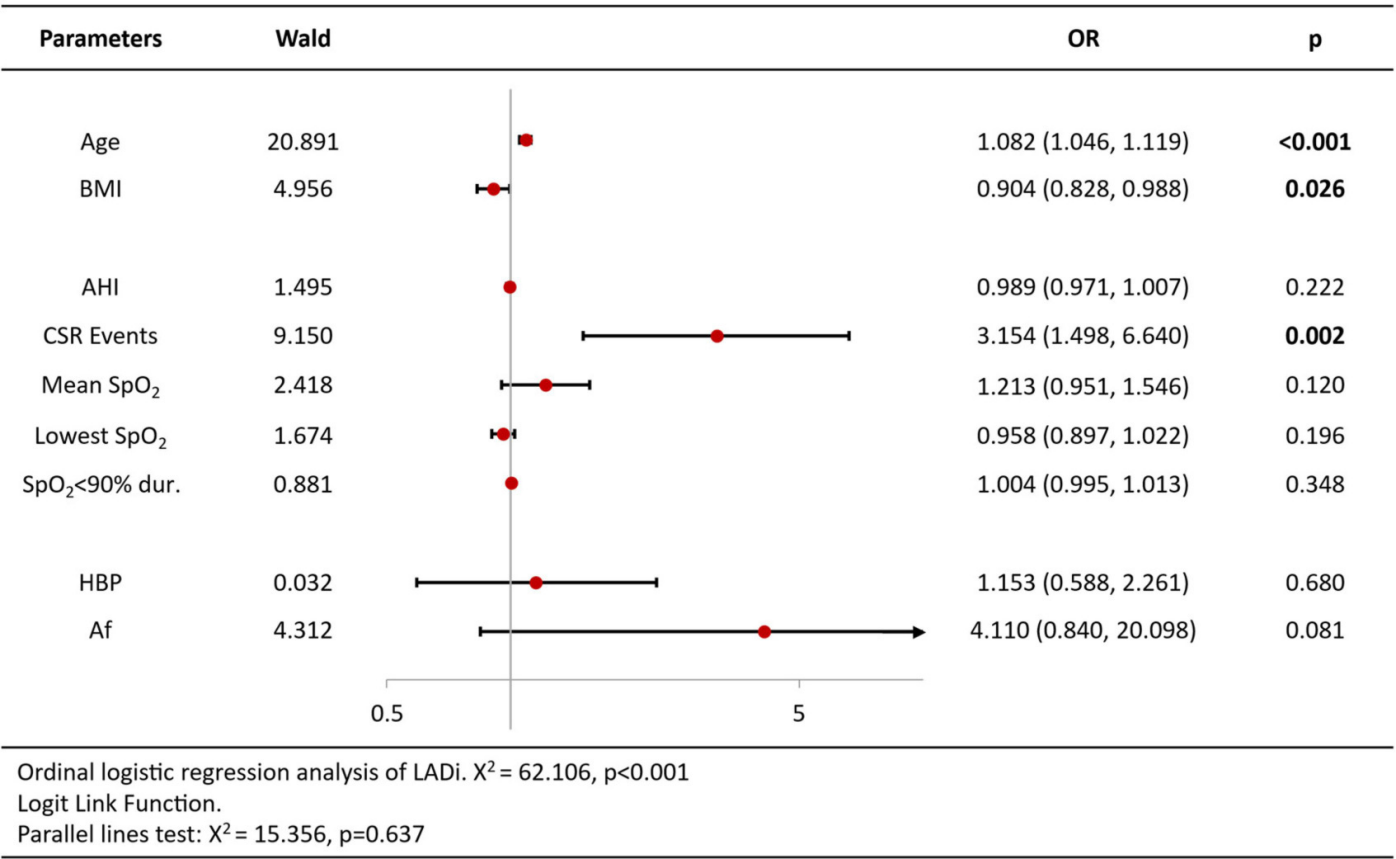			

**Table 6:** Ordinal logistic regression analysis results of average E/e’ ratio.

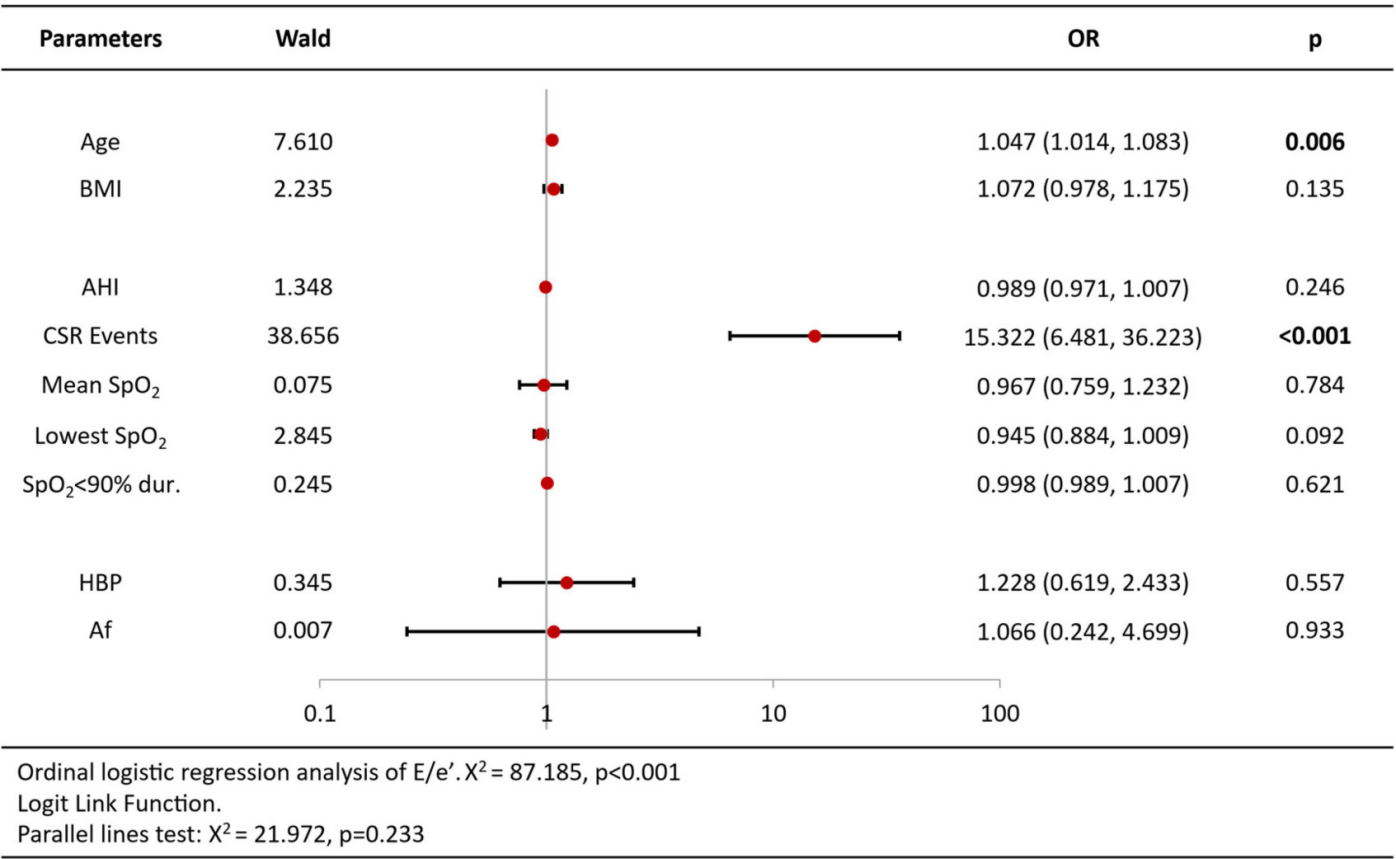			

Further linear regression analyses explored associations between CSR/TST% and cardiac markers previously identified as significant in ordinal logistic regression in Group SBD + CSR. CSR/TST% showed positive linear relationships with NT-proBNP (*β* = 0.40, *R*² = 0.16, *p* = 0.006, Supplementary Figure 1), LADi (*β* = 0.30, *R*² = 0.09, *p* = 0.047, Supplementary Figure 2), and average E/e' ratio (*β* = 0.41, *R*² = 0.17, *p* = 0.005, Supplementary Figure 3).

Binary logistic regression for IVSd abnormality (Female >1.0 cm/Male >1.1 cm) identified BMI [OR = 1.18, 95%CI (1.04–1.35), *p* = 0.013], hypertension [OR = 3.93, 95%CI (1.71–9.00), *p* = 0.001] and atrial fibrillation [OR = 0.19, 95%CI (0.04–0.98), *p* = 0.047] as significant predictors (Supplementary Table 2). For LVPW abnormality (same thresholds), age [O = 1.10 per year, 95%CI (1.01–1.10), *p* = 0.002] and BMI [OR = 1.25, 95%CI (1.09–1.44), *p* = 0.017] showed significant associations (Supplementary Table 3). Both models passed the Hosmer-Lemeshow test (*p* > 0.05) with Omnibus Tests confirming superiority over null models (*p* < 0.001). In contrast, the PA abnormality (>2.6 cm) analysis showed non-significant Omnibus Tests (*p* = 0.204) despite acceptable goodness-of-fit (Hosmer-Lemeshow *p* > 0.05), indicating insufficient evidence for a valid predictive model (Supplementary Table 4).

## Discussion

This study is the first to specifically investigate patients exhibiting CSR events without confirmed heart failure diagnosis or heart failure symptoms. We aim to explore potential associations between CSR events and cardiac structural/functional parameters in this population, with the objective of determining whether CSR may indicate specific cardiac remodeling patterns, or serve as a preclinical marker for impending heart failure. The results demonstrated that those patients exhibited older age, elevated BNP levels, higher AF prevalence, and distinct cardiac remodeling patterns featuring left atrial enlargement and increased E/e’ ratio.

Prior investigations (3, 5, 17) consistently report advanced age and predisposition to left atrial enlargement in HF patients exhibiting CSR. Our study extends these observations to non-HF individuals. Although the more sensitive parameter of left atrial volume index (LAVi) was not utilized, ordinal logistic regression models adjusted for established LADi determinants demonstrated that CSR remains independently associated with LA enlargement [including age and BMI (18, 19)].

The pivotal finding of our study demonstrates significant positive correlations between CSR events and both NT-proBNP levels and E/e', with the latter showing stronger clinical relevance. As a validated hemodynamic marker of diastolic dysfunction, E/e' synergizes with elevated BNP/NT-proBNP and LA enlargement to form a diagnostic triad for early cardiac decompensation (15, 20). Notably, the co-occurrence of these parameters—even in patients with preserved left ventricular ejection fraction (LVEF ≥50%) and normal ventricular dimensions—may signal preclinical heart failure with preserved ejection fraction (HFpEF) (15, 21). While prior research linked SBD and CSR to diastolic impairment in confirmed heart failure populations (17), our novel contribution reveals that: (1) These cardiac anomalies exhibit robust associations with CSR in non-HF individuals and (2) patients with CSR during 2-year follow-up appear to be more likely to develop confirmed heart failure clinically. This work pioneers the hypothesis that CSR may serve as a dynamic biomarker of progressive diastolic stress, marking the transition from pre-HF to overt HF or HFpEF. Given the diagnostic challenges posed by the clinically silent nature of early HFpEF, CSR detection could provide critical phenotyping clues. The temporal sequence from CSR onset to HFpEF diagnosis requires validation through multicenter prospective studies incorporating continuous cardiopulmonary monitoring.

From a pathophysiological perspective, existing studies primarily attribute the pathogenesis of CSR to abnormalities in pulmonary circulation among cardiac patients (22, 23): insufficient pulmonary blood perfusion leads to ventilation/perfusion mismatch, which subsequently establishes a periodic breathing pattern through the chemoreceptor-respiratory center feedback loop. Additionally, atrial fibrillation and progressive left atrial structural remodeling (dilation and fibrosis) reduce cardiac output, inducing circulatory delay and enhanced chemosensitivity, ultimately triggering CSR (24). This study proposes a novel mechanism whereby diastolic dysfunction triggers CSR events: in individuals with essentially normal cardiac structure, postural changes during sleep may exacerbate the adverse effects of diastolic dysfunction on pulmonary circulation hemodynamics, thereby inducing CSR episodes. This discovery opens new avenues for early cardiac function assessment research.

Furthermore, we observed that patients with concomitant sleep apnea demonstrated elevated levels of BMI, hypertension prevalence, interventricular septum thickness, and left ventricular wall thickness compared to those without sleep apnea. Subsequent statistical analysis of our longitudinal data revealed that interventricular septum thickness and left ventricular wall thickness showed stronger correlations with BMI, hypertension prevalence, and age—a pattern aligning with previous findings regarding the association between hypertension and SBD (25), as well as its impact on cardiac structural remodeling (26). These findings substantiate the association between sleep apnea, hypertension, cardiac remodeling. Although our study detected only hypertension-associated cardiac structural alterations in SBD patients without CSR, the future progression warrants attention. Untreated OSA may perpetuate cardiac injury, while respiratory control instability—manifested as elevated loop gain following CPAP therapy—could precipitate new-onset CSA or even CSR (27). These potential developments demand clinical vigilance.

## Conclusion

This study pioneers the investigation of patients exhibiting CSR events without confirmed HF diagnosis. Key findings demonstrated that individuals with CSR events presented with advanced age, elevated BNP/Nt-proBNP levels, and echocardiographic characterized by left atrial dimension enlargement and increased E/e' ratio. Multivariable regression models adjusting for potential confounders revealed independent associations between CSR occurrence and BNP/NT-proBNP levels, LADi, and E/e' ratio. Further analysis established significant positive linear correlations between CSR/TST% and NT-proBNP levels, LADi, and E/e'. These findings collectively suggest that CSR events may serve as dynamic biomarkers of progressive left ventricular diastolic dysfunction, potentially functioning as an early warning signal for the pathological transition from subclinical diastolic impairment to overt HF or HFpEF.

## Limitation

There were some limitations that should be considered when interpreting the results. First, although studies have demonstrated good inter-observer agreement for LAD, LADi, and LAVi measurements, LAVi has been shown to be more accurate than LADi in evaluating left atrial dimensions. To better monitor disease progression, LAVi assessment remains essential in subsequent studies. Besides, the retrospective and cross-sectional design of this study constrains our understanding of the association between SBD, CSR, age, AF, HF, and echocardiographic changes. A longitudinal study is warranted to follow up with enrolled patients for elucidating the specific mechanisms and temporal processes underlying the relationship between CSR and cardiac structural remodeling.

## Data Availability

The original contributions presented in the study are included in the article/Supplementary Material, further inquiries can be directed to the corresponding author.
