# Supplementary material for: Positive association between Cheyne-Stokes respiration events and diastolic dysfunction in pre-heart failure: a cross-sectional study with longitudinal implications
Source: Front Cardiovasc Med. 2025 Sep 25;12:1607079. doi: 10.3389/fcvm.2025.1607079 (PMC12507810; doi:10.3389/fcvm.2025.1607079)
Supplement: Supplementary file 1 [file Datasheet1.pdf]

Table S 1 Number of cases (n) by BNP level categories across study groups.

	**SBD+CSR (n=45)**	**SBD-CSR (n=85)**	**nonSBD (n=41)**	
Normal range[1]	22 (48.9%)	69 (81.2%)	34 (82.9%)	SBD+CSR < (SBD-CSR , NoSBD)
Mildly elevated[2]	12 (26.7%)	13 (15.3%)	5 (12.2%)	-
Significantly elevated[3]	11 (24.4%)	3 (3.5%)	2 (4.9%)	SBD+CSR > (SBD-CSR , NoSBD)

1. Normal range (BNP<35pg/mL, NT-proBNP<125pg/mL);

2. Mildly elevated (35pg/mL≤BNP<100pg/mL, 125pg/mL≤NT-proBNP<300pg/mL);

3. Significantly elevated (100pg/mL≤BNP<500pg/mL, 300pg/mL≤NT-proBNP<450-1800pg/mL with age-stratified thresholds).

SBD+CSR = Sleep breathing disorder patients with CSR Events; SBD-CSR = Sleep breathing disorder patients without CSR Events; NonSBD = Patients without Sleep breathing disorder.

Table S 2 Logistic regression analysis results of IVSd.

Parameters	Wald	OR	p
Age	2.847	1.037 (0.994, 1.081)	0.092
BMI	6.174	1.182 (1.036, 1.348)	**0.013**
AHI	0.447	1.008 (0.984, 1.033)	0.504
CSR Events	0.559	1.481 (0.529, 4.144)	0.455
Mean SpO_2	0.239	0.927 (0.686, 1.254)	0.625
Lowest SpO_2	0.244	0.980 (0.906, 1.061)	0.621
SpO_2<90% dur.	0.000	1.000 (0.987, 1.013)	0.984
HBP	10.437	3.925 (1.712, 8.997)	**0.001**
Af	3.955	0.192 (0.038, 0.976)	**0.047**

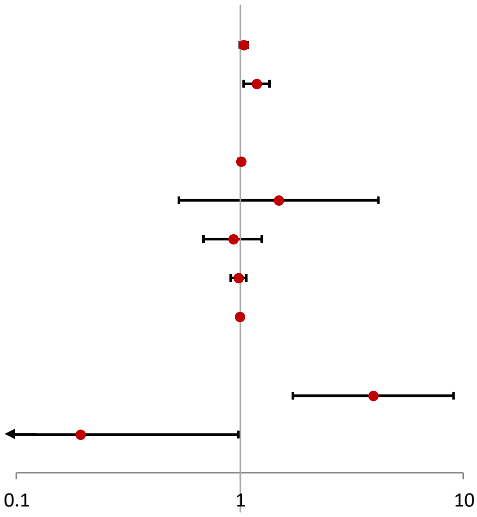

Logistic regression analysis of IVSd. Omnibus test: X^2 = 45.423, p<0.001
Hosmer-Lemeshow test: X^2 = 5.144, p=0.742

Table S 3 Logistic regression analysis results of LVPW.

Parameters	Wald	OR	p
Age	5.718	1.054 (1.010, 1.101)	**0.002**
BMI	9.669	1.251 (1.086, 1.441)	**0.017**
AHI	0.013	1.019 (0.992, 1.046)	0.170
CSR Events	0.003	0.972 (0.350, 2.695)	0.956
Mean SpO_2	0.477	1.115 (0.819, 1.517)	0.490
Lowest SpO_2	0.863	0.959 (0.879, 1.047)	0.353
SpO_2<90% dur.	0.550	0.996 (0.985, 1.007)	0.459
HBP	3.834	2.263 (0.999, 5.125)	0.050
Af	0.742	0.448 (0.072, 2.788)	0.389

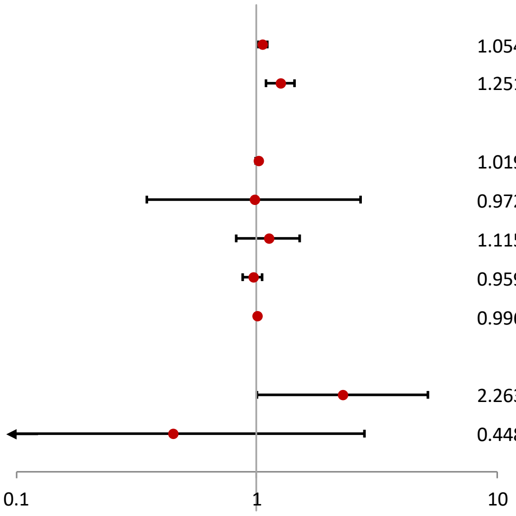

Logistic regression analysis of LVPW. Omnibus test: X^2 = 39.021, p<0.001
Hosmer-Lemeshow test: X^2 = 5.056, p=0.752

Table S 4 Logistic regression analysis results of PA.

Parameters	Wald	OR	p
Age	6.044	1.102 (1.020, 1.190)	0.014
BMI	0.095	1.029 (0.857, 1.236)	0.758
AHI	0.307	0.988 (0.947, 1.030)	0.580
CSR Events	0.180	1.391 (0.303, 6.385)	0.671
Mean SpO_2	0.151	0.899 (0.526, 1.537)	0.698
Lowest SpO_2	0.392	0.961 (0.847, 1.090)	0.531
SpO_2<90% dur.	0.051	1.002 (0.985, 1.018)	0.821
HBP	0.010	0.909 (0.134, 6.166)	0.922
Af	0.156	0.582 (0.040, 8.516)	0.693

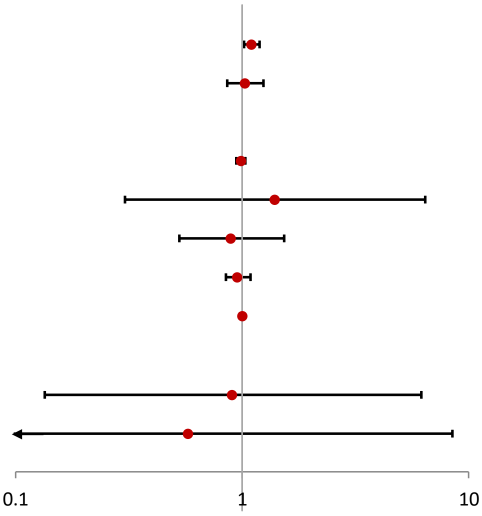

Logistic regression analysis of PA. Omnibus test: X^2 = 12.171, p=0.204
Hosmer-Lemeshow test: X^2 = 10.684, p=0.220

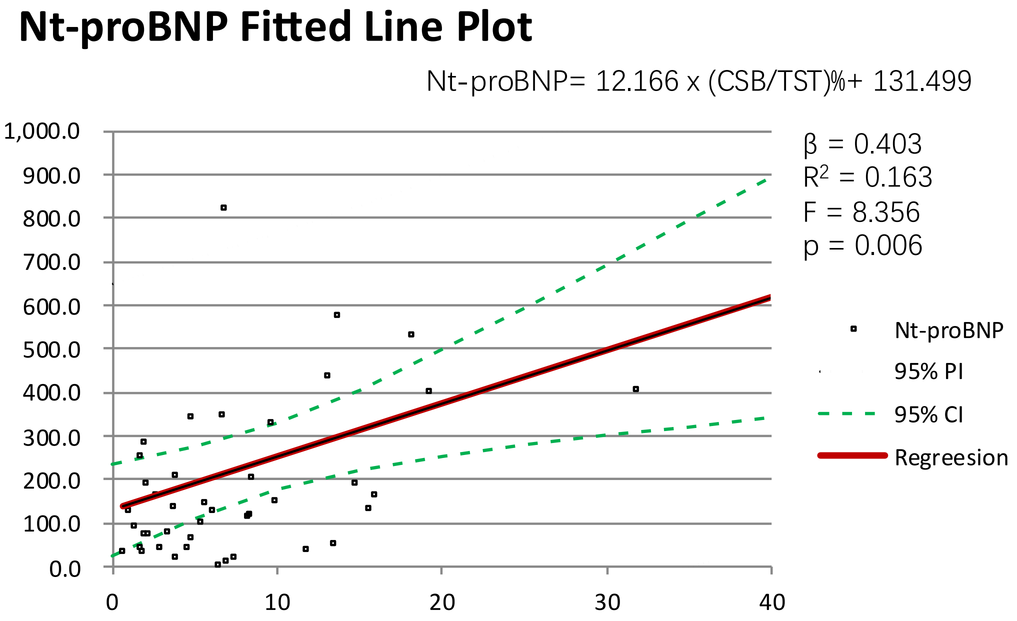

Figure S 1 Regression analysis results of Nt-proBNP in Group SBD+CSR (n=41).

A logarithmic transformation model was applied: log(NT-proBNP) = 1.1 × log(BNP) + 0.570.

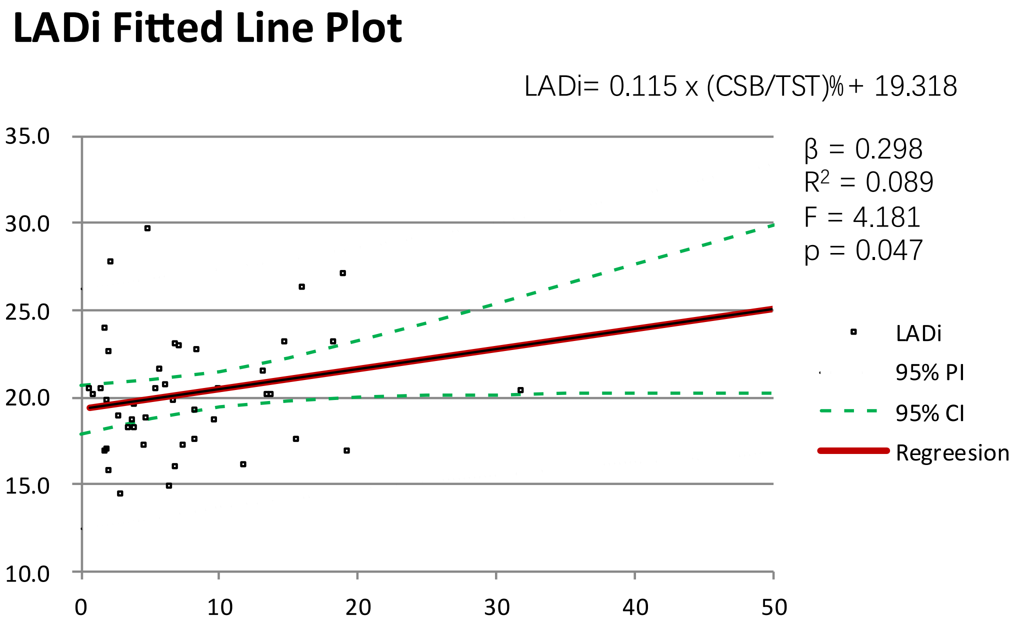

Figure S 2 Regression analysis results of LADi in Group SBD+CSR (n=41).

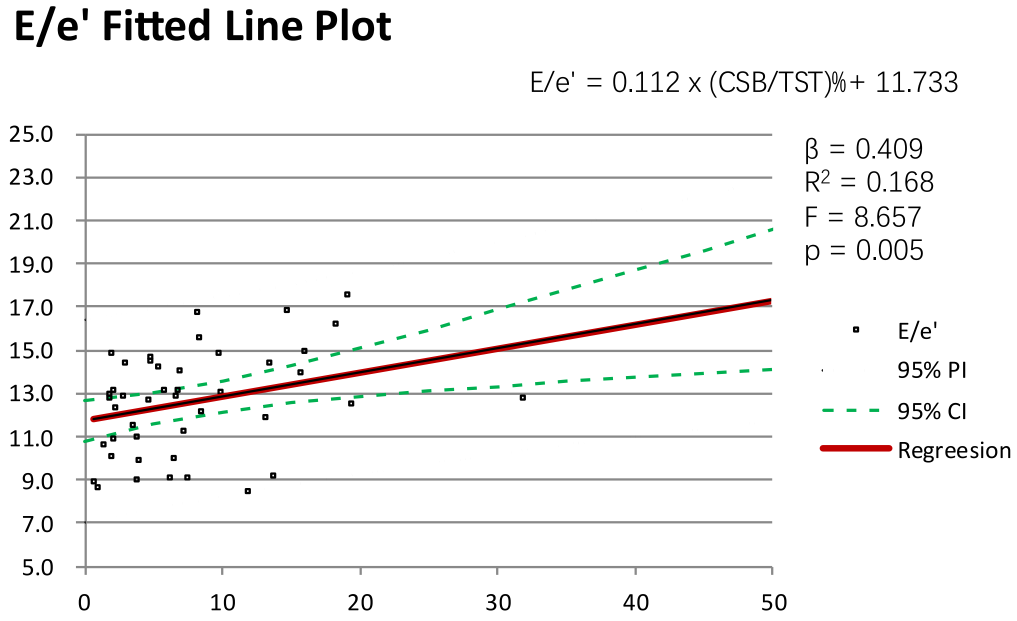

Figure S 3 Regression analysis results of E/e' in Group SBD+CSR (n=41).
